# Assessment of the LeadCare® Plus for Use on Scandinavian Brown Bears *(Ursus arctos)*

**DOI:** 10.3389/fvets.2019.00285

**Published:** 2019-08-28

**Authors:** Amanda H. Boesen, Alexandra Thiel, Boris Fuchs, Alina L. Evans, Mads F. Bertelsen, Ilia Rodushkin, Jon M. Arnemo

**Affiliations:** ^1^Department of Forestry and Wildlife Management, Faculty of Applied Ecology, Agricultural Sciences and Biotechnology, Inland Norway University of Applied Sciences, Koppang, Norway; ^2^Center for Zoo and Wild Animal Health, Copenhagen Zoo, Frederiksberg, Denmark; ^3^ALS Scandinavia AB, Luleå University of Technology, Luleå, Sweden; ^4^Department of Wildlife, Fish and Environmental Studies, Faculty of Forest Sciences, Swedish University of Agricultural Sciences, Umeå, Sweden

**Keywords:** blood lead, lead exposure, *Ursus*, anodic stripping voltammetry, Pb

## Abstract

Lead (Pb) exposure is associated with adverse health effects in both humans and wildlife. Blood lead levels (BLL) of sentinel wildlife species can be used to monitor environmental lead exposure and ecosystem health. BLL analyzers, such as the LeadCare®, are validated for use in humans, assessed for use in some avian species and cattle, and are increasingly being used on wildlife to monitor lead exposure. The LeadCare® analyzers use a technique called anodic stripping voltammetry (ASV). Species-specific conversion equations have been proposed to approximate the levels found with gold standard measuring methods such as inductively coupled plasma mass spectrometry (ICP-MS) because the ASV method has been shown to underestimate BLL in some species. In this study we assessed the LeadCare® Plus (LCP) for use on Scandinavian brown bears (*Ursus arctos*). LCP measurements were correlated with ICP-MS with a Bland-Altman analyzed bias of 16.3–22.5%, showing a consistent overestimation of BLL analyzed with LCP. Based on this analysis we provide conversion equations for calculating ICP-MS BLL based on the LCP results in Scandinavian brown bears. Our study shows that the LeadCare® Plus can be used for monitoring of lead exposure by approximating gold standard levels using conversion equations. This enables comparison with other gold standard measured BLL within the observed range of this study (38.20–174.00 μg/L). Our study also found that Scandinavian brown bears are highly exposed to environmental lead.

## Introduction

Environmental contamination with and subsequent exposure to lead (Pb) impacts both humans and animals living in a polluted ecosystem. In humans lead has been associated with a wide range of harmful health effects, including reduced IQ (1) and cardiovascular disease (2, 3). The World Health Organization states that there are no safe levels of lead in the body (4) and even low level exposure poses a health risk, especially during developing stages (5). Although the harmful effects of lead exposure in humans are well-documented, these results have yet to be applied in legislation and regulation on all areas of lead usage, e.g., lead-based ammunition for hunting. The European Food Safety Authority (EFSA) has set the blood lead level (BLL) of concern for developmental neurotoxicity in humans to 12 μg/L (6). The level of concern of the American Centers for Disease Control and Prevention (CDC), now termed the “reference level” under the current US administration, is 50 μg/L (7, 8).

Lead is absorbed through ingestion and inhalation (9), with higher absorption from the gastrointestinal tract in children than in adults (10). Lead can be measured in the blood (11). Lead compounds are inorganic, organic or ionic, where organic lead can be metabolized to inorganic and ionic lead (12). Ionic lead exerts similar toxicities as inorganic lead. As a bio accumulative toxicant, 94% of the lead body burden is stored in bone (13). The half-life for inorganic lead is 30 days in blood and 10–30 years in bone (6, 10). The half-life seems to be positively correlated to the length of exposure (14) and varies with age and sex (15).

Lead follows a three-compartment distribution model between blood, soft tissue and bone (11). Lead is excreted via urine and feces as well as in hair, sweat and nails (11). BLL can be used to measure recent exposure, but bone-stored lead can be reabsorbed and become an endogenous source of lead, keeping the blood level elevated. Increased calcium mobilization through bone resorption and concurrent reabsorption of lead to the blood is seen during pregnancy and lactation in humans (16), with highest mobilization of bone stored lead in the postpartum period (17). This causes prenatal and neonatal exposure through the placenta (18) and nursing in humans (19) and mice (20). The lactose content of the milk promotes calcium absorption in the gut, and thereby also increases lead absorption (21).

The gold standard of lead analysis in blood or tissue is generally accepted to be graphite-furnace atomic absorption spectrometry (GFAAS) or inductively coupled plasma mass spectrometry (ICP-MS) (22). ICP-MS and GFAAS has been determined to be statistically equivalent when measuring BLLs for clinical use and biomonitoring (23, 24). Both methods are expensive and time-consuming and require laboratory involvement. An alternative analytical method that both simplifies the process and lowers the cost of determining BLL has been introduced: Anodic stripping voltammetry (ASV) is a relatively new method that uses electrochemistry to measure lead in blood (25).

The LeadCare® analyzers from Magellan Diagnostics Inc. (North Billerica, MA, USA) apply ASV technology. These analyzers are validated for humans (26), some avian species (27, 28) and in cattle (29) by comparison to the GFAAS and/or ICP-MS. Furthermore, ASV has been used in studies investigating lead exposure in grizzly bears (*Ursus arctos*), black bears (*U. americanus*), gray wolves (*Canis lupus*) and cougars (*Puma concolor*) (30), and multiple avian species (31, 32). The analyzers are portable and require little training to operate. This is beneficial in wildlife research and biomonitoring of lead exposure in the field.

In studies comparing the LeadCare® systems with GFAAS or ICP-MS, the ASV analyzers had a negative bias (i.e., underestimation) when measuring the BLL of avian wildlife (27, 33) and cattle (29). Validation for each species has been proposed to verify the conversion rate when using the LeadCare® to monitor exposure (23). No significant difference was found between the different LeadCare® systems in birds (23). In the present study, the LeadCare® Plus (LCP) was used as it has the lowest detection range of the currently available LeadCare® analyzers.

The objective of this study was to compare LCP and ICP-MS for measurement of BLL in Scandinavian brown bears. Based on previous studies on hunting with lead-based ammunition in Scandinavia (34), the scavenging behavior of Scandinavian brown bears (35) and assessment of the LeadCare® system in other species, we predicted (1) to find detectable levels of lead in blood of some bears and (2) to find that LCP was negatively biased when compared to ICP-MS.

## Methods and Materials

The present study was conducted in Dalarna and Gävleborg counties in south-central Sweden (61°N 15°E) and Hedmark County in south-eastern Norway (61°N 18°E) as part of the Scandinavian Brown Bear Research Project (36). Captures, handling and sampling of bears were approved by the Swedish Ethical Committee on Animal Research (Uppsala, Sweden; #C18/15), the Swedish Environmental Protection Agency (Stockholm, Sweden; NV-0758-14), and the Swedish Board of Agriculture (#31-11102/12) and were carried out according to an established protocol (37).

Blood was collected from the jugular vein in 4 ml EDTA tubes using the Vacuette® system (Greiner Bio-One, Kremsmünster, Austria) from 54 bears captured during April-May 2018. The blood samples were analyzed on the LCP according to the protocol for the analyzer, either within 6 h of sampling, or after refrigeration at 4°C for up to 72 h. In addition, 70 frozen samples stored at −20°C for 1 to 8 years (2010: *N* = 11, 2013: *N* = 23, 2017: *N* = 36) were analyzed. The frozen samples were thawed at room temperature and inverted to homogenize the content before being tested on the LCP with the same method as the fresh blood samples.

Fifty microliter whole blood was transferred using a calibrated autopipette (Eppendorf Research® 10–100 μL, pipette tips 10–100 μL, Eppendorf, Hamburg, Germany) into a test vial provided in the test kit for the analyzer. Test vials contain hydrochloric acid solution that breaks down the red blood cells. The vial was inverted 8–10 times, then stored out of sunlight at room temperature for 24 h. After 24 h, the test vial was inverted 8–10 times and 30 μL was transferred with the autopipette to the test strip of the analyzer. The analysis took 3 min and the result was provided in μg/dL. The analyzer does not have memory, so the result was written down before the used sample strip was removed.

For each day of analysis with the LCP a quality control set from the test kit was analyzed. All quality controls analyzed during this study were within the control range given for the respective test kits used.

After analysis on the LCP, the samples were stored at −20°C in the collection tubes. The samples were then sent for analysis at ALS Scandinavia AB (Luleå, Sweden) to determine the BLL with ICP-MS. The freezing chain was not further interrupted. The samples were thawed, inverted for several minutes, and an aliquot of whole blood was digested with concentrated nitric acid (closed vessel, MW-assisted digestion using Mars5 laboratory digestion system from CEM) followed by dilution with distilled, de-ionized water. Diluted digests were analyzed by ICP-SFMS (ELEMENT2, ThermoScientific, Bremen, Germany), using combination of internal standardization and external calibration. Details on preparation method, measuring parameters and operation conditions can be found elsewhere (38, 39). Method blanks prepared and analyzed alongside with blood samples contain <0.5 μg/L Pb. Accuracy of the method in Pb concentration range 10–460 μg/L was controlled using reference materials from Sero AS (Oslo, Norway, Seronorm Trace elements in Human Whole Blood, Lot 1702826A, L1, L2, and L3) reconstituted according to the manufacturer's instructions.

### Statistical Method

All statistical analyses were carried out with R (40). Linear regression modeling was used to assess the agreement between the LCP and the ICP-MS concentrations. BLL_ICP−MS_ was modeled as response with BLL_LCP_ as explanatory variable. Regression coefficients of the model outputs were used to develop conversion equations. The LCP and the ICP-MS BLLs were log transformed to improve normality of error and distribution.

The BLLs were analyzed both pooled and separated into fresh and frozen. One sample was excluded as an extreme outlier with BLL_LCP_ > 650 μg/L (over detection limit of the analyzer) and BLL_ICP−MS_ = 174 μg/L. The final sample size was *n* = 124 with 54 fresh and 70 frozen samples. The LCP concentration in μg/dL was converted to μg/L. As quantity of frozen samples from each sample year was not enough to analyse each year statistically, all frozen samples were analyzed as one category.

Previous wildlife studies have identified a bias of the ASV method, so Bland-Altman method comparison analysis (41) using R package *blandr* (42) was used to determine a potential bias on BLL of Scandinavian brown bears between the analysis methods of ASV and ICP-MS.

Models tested on log-transformed data:

$$\begin{array}{l} \text{Model }1\;BLL_{ICP-MS\;pooled}=\beta_{0}+\;\beta_{1}\left(BLL_{LCP\;pooled}\right)+\;\epsilon \\ \text{Model }2\;BLL_{ICP-MS\;fresh}=\beta_{0}+\;\beta_{1}\left(BLL_{LCP\;fresh}\right)+\;\epsilon \\ \text{Model }3\;BLL_{ICP-MS\;frozen}=\beta_{0}+\;\beta_{1}\left(BLL_{LCP\;frozen}\right)+\;\epsilon \end{array}$$

Significance for analysis was set at α = 0.05. Diagnostics plots for the regression models were checked.

## Results

BLL analyzed with the LCP had a median of 99 μg/L (mean 106.3 μg/L) in a range of 40–231 μg/L. BLL analyzed with ICP-MS had a mean of 88.1 μg/L (median 83 μg/L) in a range of 32.5–173.0 μg/L.

**Figure 1:**
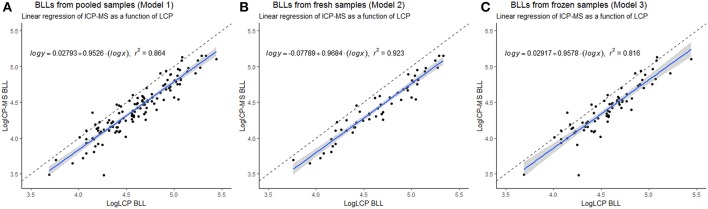
Linear regression of pooled, Model 1 **(A)**, fresh, Model 2 **(B)**, and frozen, Model 3 **(C)** blood lead levels (BLL) of Scandinavian Brown bear (*Ursus arctos*) (sampled 2010–2018) measured with inductively coupled plasma mass spectrometry (ICP-MS) as a function of BLL measured with LeadCare® Plus (LCP). Linear regression model (blue) with 95% confidence interval (gray) and added dashed line of optimal 1 to 1 comparison, and equation of the model with *r*^2^. Plotted with log transformed data.

**Figure 2:**
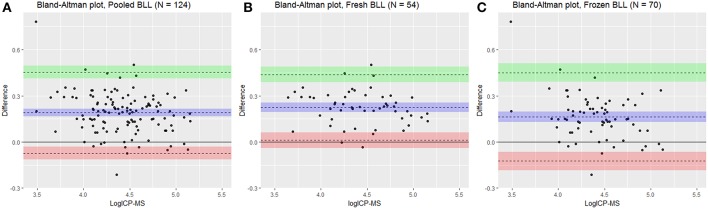
Bland-Altman plot for log transformed pooled **(A)**, fresh **(B)**, and frozen **(C)** blood lead levels (BLL) in Scandinavian brown bear *(Ursus arctos*) comparing analysis methods Inductively coupled plasma mass spectrometry (ICP-MS) and LeadCare® Plus (LCP). X axis shows the log ICP-MS as gold standard reference and Y axis shows differences (logBLL_LCP_-logBLL_ICP−MS_). Represented is mean difference (bias) in blue line with 95% confidence interval in shaded blue with upper limit of agreement of Bland-Altman analysis in green line with 95% confidence interval in shaded green (+1.96^*^standard deviation) and lower limit of agreement (−1-96^*^standard deviation) in red line with 95% confidence interval in shaded red.

The LCP was significantly correlated to the ICP-MS BLL both for overall as well as fresh and frozen samples separately (Table 1, Figure 1) and conversion equations were based on the models 1, 2 and 3. An overall positive bias of the LCP was found with Bland-Altman analysis. For pooled samples, 19.01% (CI: 16.62–21.40%), for fresh samples at 22.50% (CI: 19.53–25.47%), and for frozen samples 16.32% (CI: 12.83–19.82%) (Figure 2) showing an overestimation of the BLL compared to ICP-MS results.

**Table 1:** Model parameters with *P*-values, *r*^2^ and Bland-Altman analyzed bias showing a positive bias of the LeadCare® Plus (LCP) for pooled (*N* = 124), fresh (*N* = 54), and frozen (*N* = 70) samples of blood lead levels in Scandinavian brown bear (*Ursus arctos*) and conversion equation based on the regression coefficients for the models 1, 2, and 3 to estimate blood lead levels when analyzed with Inductively coupled plasma mass spectrometry (ICP-MS).

**ICP-MS as a function of LCP**	***P***	***r^**2**^***	**Bland-Altman bias [CI95]**	**Conversion equation**
Model 1—Pooled	<0.001	0.864	0.1901 [0.1662–0.2140]	logy = 0.0279 + 0.9527(log x)
Model 2—Fresh	<0.001	0.923	0.2250 [0.1953–0.2547]	logy = −0.0779 + 0.9684(log x)
Model 3—Frozen	<0.001	0.817	0.1632 [0.1283–0.1982]	logy = 0.0292 + 0.9579(log x)

*CI95: 95% confidence interval*.

When applying the conversion equation on the BLL_LCP_, the new, calculated BLL was not significantly different from the BLL_ICP−MS_ (pooled, *P* = 0.933; fresh, *P* = 0.978; frozen, *P* = 0.988) with similar range.

## Discussion

The results showed a high correlation between LCP and ICP-MS, although with a linear positive bias (overestimation) of 16.3–22.5% of the LCP. Conversion equations for BLL_LCP_ were established to estimate BLL_ICP−MS_ in brown bears for the observed range of this study. Furthermore, the results indicate that Scandinavian brown bears are exposed to elevated BLL.

The positive bias of the LCP contrasts with all other comparison studies conducted on wildlife and cattle, as these studies found a negative bias on the analyzers using the same method (ASV). The frozen samples of our study had a smaller bias when compared to the gold standard which is in alignment with findings by Herring et al. (23) in avian species. Conversion equations for the LCP results to estimate ICP-MS BLL both for pooled, fresh and frozen samples were made, which can be used for future studies in brown bears to approximate the gold standard levels. The pooled conversion formula was provided for the use with a small sample size comprising of both fresh and frozen samples. The higher *r*^2^-value of the pooled conversion formula could be influenced by the larger sample size in this group.

The BLLs of this study were higher than the levels Rogers et al. (30) found in black bears and grizzly bears in Yellowstone, however these levels were only analyzed on a LeadCare® system, and were not validated.

BLLs cannot be directly compared with levels measured using other methods because of the identified bias. Using LCP BLL without correcting for the bias could lead to an overestimation of the actual lead exposure within the observed range of this study.

### Factors Affecting BLL or Measurements

The LeadCare® manufacturer recommends to use fresh blood samples, and the FDA permits the use of the machine when used for capillary samples for humans (43). Freezing has been shown to impact the results of BLLs in wildlife (23) and humans (44), and the length of freezing may have had an effect on the measured BLL in this study as the freezing causes evaporation. We saw a smaller bias between the LCP and the ICP-MS in our samples that had been stored frozen prior to LCP analysis, with cause yet to be identified. To examine possible evaporation due to freezing, the samples could in future analysis be weighed before freezing and after thawing for reanalysis after long term storage.

Packed cell volume (PCV) could affect the measured BLLs, as stress throughout the capture, and especially during the chasing period, can cause dehydration and contraction of the spleen during exertion (45). A higher PCV likely gives a higher BLL reading using any analysis method, because of a relatively higher concentration of red blood cells, to which a majority of the lead is bound. Reference PCV for free-ranging brown bears is 0.41–0.54% (46) following the same capture methods as used in this study, with a seasonal variation of higher PCV in winter compared to summer (47, 48).

BLLs show recent lead exposure or endogenous lead remobilised due to bone resorption. During hibernation, the bear maintains bone mass through balanced bone resorption and formation activity (49, 50). Continuous endogenous exposure could keep the BLLs elevated during hibernation and possibly result in the high concentrations observed in the spring.

A trend of higher bone resorption is seen in bears that give birth (51). As lead is transferred through the placenta and through the milk during the lactational period (18), there could be a higher endogenous exposure in females but overall lower body lead burden because of higher excretion compared to males. The accumulative body lead burden can be measured in bone to investigate life history exposure and sex differences of the bears. However, accumulation site depends on bone formation activity at time of exposure (52). This is in alignment with findings by Herring et al. (53) that measured bone lead in the femoral bone of brown bears in Croatia. They found differing concentrations within the same individual, dependent on location of sampling site for both trabecular and compact bone.

The LeadCare® analyzers were recalled in May 2017 (54) due to underestimations of BLLs when using venous blood samples in humans. Caldwell et al. (55) reported that the CDC quality assurance program in the last five quality challenges found that participating laboratories were able to accurately analyse BLLs with a LeadCare® Ultra or Plus in 70% of the cases with a set evaluation criteria limit of ±20 μg/L on bovine venous samples that had been stored frozen until distribution. In the same study, overall accuracy of all participating laboratories with no distinction between methods was 89% with the same evaluation criteria limit of ±20 μg/L. This inaccuracy is to be kept in mind when analyzing LCP results.

### Perspectives

Lead exposure from spent hunting ammunition is considered a major ecological and One Health concern (56). Ingestion of lead ammunition has not definitively been identified in bears (30). However, novel ways to identify source of exposure from other wildlife studies can be implemented, like measuring antimony (Sb) (57), another component of lead alloy in lead ammunition (58, 59). As lead is present in the environment, other food sources such as berries and ants should be investigated as potential sources, as well as exposure from active lead mines and secondary lead smelters.

The BLLs found in this study show a significant exposure to lead with unknown behavioral or physiological impact on the bears. Accurate monitoring of lead exposure in wildlife species is essential for identifying risks associated with toxicants that are proven harmful. The bear could be used as a sentinel species, as it is an opportunistic omnivore (60) and scavenger (35, 61). Using bear blood as a biomarker for recent exposure, or to compare temporal studies before and after legislative changes, is a relatively easy and readily available tool in biomonitoring.

Monitoring of acute or endogenous lead exposure in individuals, for health assessment, as well as on population level can be carried out using portable lead analyzers such as the LeadCare® Plus with provided conversion equations to approximate gold standard levels within the observed range of this study.

This is the initiating study on lead in Scandinavian brown bears and further research is necessary to investigate potential harmful effects and possible sources of exposure.

## Data Availability

The dataset for this study is available upon request.

## Author Contributions

JA: study design. AB, AT, BF, AE, and JA: capture and sampling. AB, AT, and AE: LeadCare® analysis. IR: inductively coupled plasma mass spectrometry analysis. AB, AT, and BF: data analysis. AB: drafting of manuscript. AB, AT, BF, AE, MB, IR, and JA: revision of manuscript and approval of submitted version.

### Conflict of Interest Statement

The authors declare that the research was conducted in the absence of any commercial or financial relationships that could be construed as a potential conflict of interest.
